# Primary Non-Hodgkin B Cell Lymphoma in a Man

**Published:** 2011-03-30

**Authors:** Sh. M. I. Alhabshi, Z. Ismail, Sh. A. Arasaratnam

**Affiliations:** 1 Lecturer and Consultant Radiologist, Department of Radiology, Faculty of Medicine, University Kebangsaan Malaysia Medical Center, Kuala Lumpur, Malaysia; 2 Fellowship in Breast Imaging. Consultant Radiologist, Department of Radiology, Serdang Hospital, Selangor, Malaysia; 3 Fellowship in Breast Imaging. Consultant Radiologist, Department of Radiology, Kuala Lumpur, Malaysia

**Keywords:** Breast Lymphoma, Non Hodgkin Lymphoma, Male Breast, Ultrasound, MRI

## Abstract

Malignant breast lymphoma is a rare condition and primary breast lymphoma is extremely rare in the male population. We present a case of a 26-year-old man (transgender) who presented with a large palpable mass in the right breast. This mass was rapidly growing in size associated with right axillary lymphadenopathy. Ultrasound and MRI findings were consistent with BIRADS IV lesion which was suspicious of malignancy. Core biopsy was performed and histopathology confirmed the diagnosis of primary non Hodgkin B cell lymphoma of the breast.

## Introduction

Non-Hodgkin's primary breast lymphoma (PBL) is a very rare occurrence. The incidence of primary breast lymphoma is estimated to be about 0.04-0.5% of all lymphomas.[1] The diagnosis of primary breast lymphoma is limited to patients who have no evidence of systemic lymphoma or leukemia when the breast lesion is detected. Secondary breast lymphoma with extra mammary involvement occurs more frequently and commonly presented with multiple breast lesions. Nevertheless, in secondary breast lymphoma the patient is usually a known case of lymphoma with concurrent breast lesion associated with one or more non-mammary organs.[2] Primary MALT-type lymphoma is a specific subset of non-Hodgkin lymphoma which may become disease-free after local therapy. Secondary breast lymphoma is more heterogeneous presenting with a higher grade.[3] Most of the reported cases are females. PBL is extremely rare in males. We report a case of a primary B-cell nonHodgkin’s lymphoma of the breast in a male patient with emphasis on ultrasound and MRI findings.

## Case Presentation

A 26-year-old man (transgender) with no medical illness complained of a large palpable mass in the right breast. The mass was rapidly growing in size for two months. He had been taking hormonal pills for the past five years for breast enlargement but had stopped for the past two months when he noted rapid enlargement of the right breast mass. There was no history of breast cancer or any other malignancy in the family. There were also no history of fever, night sweat and weight loss to suggest B-symptoms.

**Fig. 1:**
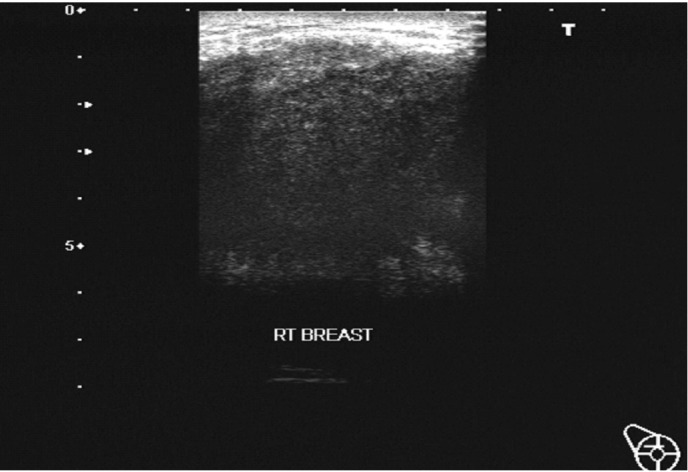
A 26-year-old transgender with primary breast lymphoma. Ultrasound of the right breast shows a large irregular predominant hypo-echoic mass with heterogeneous internal echotexture and cast posterior shadow.

On examination, there was a large and hard mass occupying the right breast measuring 6.0×5.0×4 cm. The overlying skin appeared tense. Ultrasound of the breast showed an ill-defined, hypo-echoic mass with heterogeneous internal echotexture and cast posterior shadow (Fig. 1). There were multiple ipsilateral enlarged axillary lymph nodes.

**Fig. 2:**
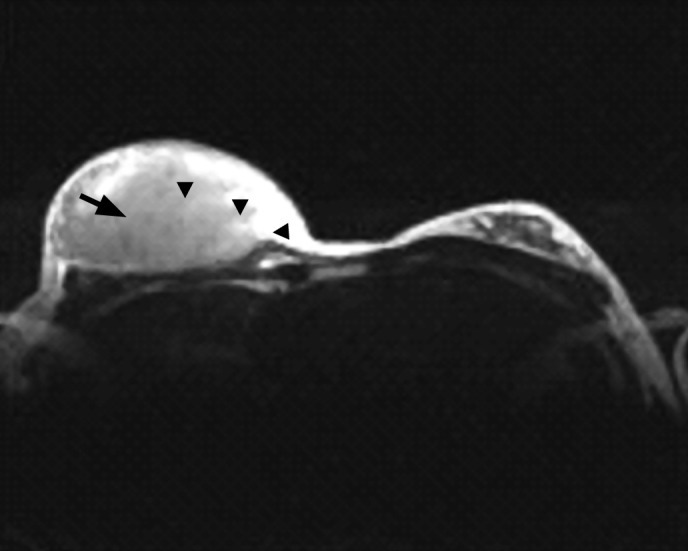
MRI axial T2 weighted image in the same patient shows the irregular mass (arrow heads) with intermediate signal intensity. There is also a dilated duct within the mass (arrow).

Magnetic resonance imaging showed a heterogeneously large globular enhancing mass occupying the right breast. This mass showed heterogeneously low signal on T1WI and high signal on T2WI (Fig. 2). There was a tubular structure which was directed toward the nipple and showed low signal on T1WI and high signal in T1WI with no significant enhancement in post-contrast images which was suggestive of an abnormal dilated duct (Fig. 2).

**Fig. 3:**
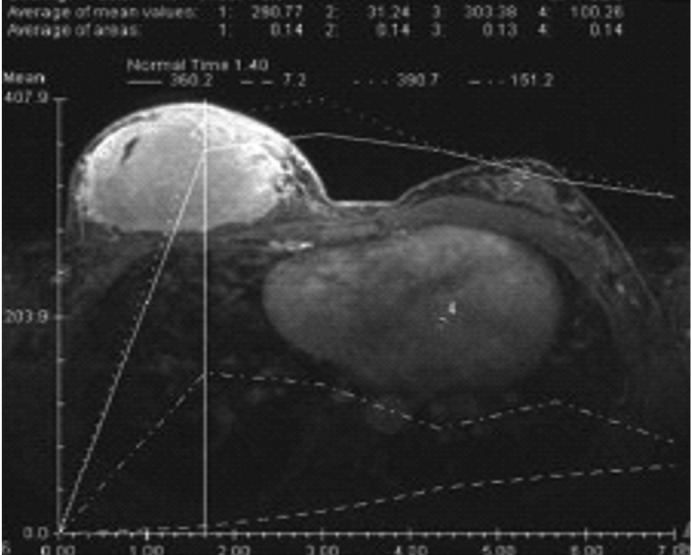
Dynamic contrast enhancement curve shows type II (plateau) pattern which is consistent with an indeterminate lesion.

Dynamic contrast enhancement showed type II curve (Fig. 3) which was consistent with an indeterminate lesion. The left breast was normal.

Radiological and clinical findings were consistent with BIRADS IV lesion which suspicious of malignancy. Core biopsy was done and histopathological examination confirmed diagnosis of B-cell type non-Hodgkin lymphoma. Computed tomography scan of the neck, thorax and abdomen, which were performed for further assessment showed no other mass or enlarged nodes. The patient was referred to the hematology unit for further management. He was started on CHOP regime of chemotherapy (adriamycin, cyclophosphamide, prednisolone, vincristine and bleomycin) for 6 cycles followed by radiotherapy (40±50 Gy) to the breast and adjacent axillary lymph nodes. The patient responded well to treatment and after two years the patient reached complete remission.

## Discussion

Primary breast lymphoma is rare in both genders and the incidence ranges from 0.12% to 0.53% of all breast malignancies.[1] Most of the reported cases are women. It is extremely rare in men as seen in this patient.

The clinical presentation and radiological features of breast lymphoma and carcinoma are similar. Both usually present as painless enlarging breast lumps. Rapid painless enlargement of the mass may also suggest the diagnosis of PBL.[1] Most of the patients will present with a palpable mass, either focal or diffusely enlarged.[3] Patients presenting with breast lump with B-symptoms like fever, night sweat and loss of weight have helpful clinical signs that indicate the need for further follow up to rule out breast lymphoma. However, this patient had no B-symptoms on presentation.[3]

PBL which relates to sex hormone has been reported.[4] The breast tissue is sex hormone dependant and lymphoid tissues in the breast are influenced by alteration of the systemic sex hormones due to several conditions particularly pregnancy or breast feeding.[4] This patient had a history of taking hormonal pills; therefore, this was probably the main factor contributing to the formation of lymphoma in the breast. Evidence also suggested that lymphoma of the breast was equivalent to the malignant lymphomas of the mucosa-associated lymphoid tissues (MALT).[3]

Ultrasound appearances of our patient were consistent with the previous reported cases of breast lymphoma. The mass is often ill-defined and heterogeneous hypoechoic.[5] Recent reviews have described nonspecific ultrasound features of breast lymphoma, including an ill-defined heterogeneously hypoechoic mass associated with overlying skin and subcutaneous edema. The rest of the ultrasound findings include well-defined hypoechoic masses or diffuse nodular infiltration of the breast.[2] Another series which described the ultrasound features of breast lymphoma stated that the most common ultrasound features for breast lymphoma were solitary hypervascular masses (64%), ill-defined margins (59%), echogenic (27%), hypoechoic (59%) or mixed hypo- and hyperechoic (23%) masses.[2] However, it is difficult to differentiate breast malignancy and lymphoma by ultrasound alone.

Lymphoma is relatively heterogeneous on MR images. They tend to be hypointense to fat on T1WI and hyperintense to muscle on T2WI. In the dynamic post-contrast sequences, the mass enhance heterogeneously and typically showed type II curve which was similar to indeterminate or suspicious of malignant masses.[2] This finding is consistent with our patient’s MRI findings.

Most patients with older series were treated with mastectomy and axillary dissection or combination with radiotherapy. Recent articles have recommended chemotherapy is the effective treatment for PBL without surgery which in 90% of instances show good response. Moreover, primary breast lymphoma (PBL) generally has better prognosis than breast carcinoma and secondary lymphoma with a 90% 5-year survival rate.[5]

Overally, we may conclude that primary breast lymphoma in contrast to breast carcinoma is extremely rare, especially in men. It should be considered as the differential diagnosis of breast mass by correlating the clinical presentation, imaging appearances as well as history of hormonal intake. Despite the clinical and radiographic similarities, the treatment options differ. For this reason, it is important to correctly differentiate lymphoma from other breast malignancies to avoid unnecessary mastectomy and axillary clearance.
